# Very long-term follow-up of aplastic anemia treated with immunosuppressive therapy or allogeneic hematopoietic cell transplantation

**DOI:** 10.1007/s00277-020-04271-4

**Published:** 2020-09-19

**Authors:** Beatrice Drexler, Felicitas Zurbriggen, Tamara Diesch, Romaine Viollier, Joerg P. Halter, Dominik Heim, Andreas Holbro, Laura Infanti, Andreas Buser, Sabine Gerull, Michael Medinger, André Tichelli, Jakob R. Passweg

**Affiliations:** 1 Division of Hematology, University Hospital, Basel, Switzerland; 2 Division of Pediatric Oncology/Hematology, University Children’s Hospital Basel UKBB, Basel, Switzerland; 3 AUREA, General Practioner office, Basel, Switzerland; 4 Blood Transfusion Center, Swiss Red Cross, Basel, Switzerland

**Keywords:** long-term outcome, aplastic anemia, immunosuppressive therapy, allogeneic hematopoietic cell transplantation

## Abstract

**Introduction:**

Since the 1970s outcome of aplastic anemia (AA) patients has improved significantly due to the introduction of immunosuppressive therapy (IST) and allogeneic hematopoietic transplantation (HCT). However, patients may suffer from persistent disease, relapse, clonal evolution, graft-versus-host disease and other late effects. Here, we analyse very long-term outcome of all AA patients at our institution comparing not only survival, but also response status and complications.

**Methods:**

Patient charts of all 302 AA patients treated between 1973 and 2017 at the University Hospital Basel, Switzerland, were retrospectively analysed.

**Results:**

First line treatment was IST in 226 (75%) and HCT in 76 (25%) patients. Overall survival at 30 years was similar in patients treated initially by HCT and IST (44% (±14%), and 40% (± 9%) respectively, with better results in more recent years. Partial and no response occurred more frequently after IST, relapse incidence after IST was 24 %, whereas non-engraftment and graft failure was documented in 15 patients (19 %) after HCT. Clonal evolution to myelodysplastic syndrome / acute myeloid leukemia was 16 % at 25 years in IST patients, 1.3 % in HCT patients, iron overload (18 versus 4 %, p = 0.002) and cardiovascular events (11 versus 1 %, p=0.011) occured significantly more often in IST than HCT treated patients. The majority of long-term survivors, 96% of those alive at 25 years, were in complete remission at last follow up, irrespective of the initial treatment modality.

**Conclusion:**

Very long term survivors after AA are those with stable hematopoietic recovery.

## Introduction

Aplastic anemia (AA) is a rare hematological disease characterized by bone marrow failure causing pancytopenia with symptoms of bleeding, anemia and infections. Originally a fatal condition, outcome has improved significantly since the 1970s, particularly due to introduction of immunosuppressive therapy (IST) with anti-thymocyte globulin (ATG) and hematopoietic cell transplantation (HCT) as the major first line treatment strategies [1, 2]. The standard regimen for first-line IST produces hematological recovery in 60-70 % of cases and shows excellent long-term survival among stable responders [3]. However, failure free survival after one course of IST is only approximately 25% [4]. HCT on the other hand is associated with high cure rates of AA reaching survival rates up to 90 % and higher in some single center studies [5], less with studies from larger registries and mostly with follow-up limited to several years. Adjustments to the treatment protocols and improvement in supportive care have increased overall survival (OS) over the last decades, recently analysed in 6293 patients from the EBMT registry of the SAA Working Party [6]. Independent of survival, patients in the IST group still may show incomplete or no hematological recovery, experience relapse in about 35 % at 5 years [7], and are at risk for late clonal disorders like myelodysplastic syndrome (MDS) or acute myeloid leukemia (AML) [8, 9]. Transplanted patients on the other hand are exposed to risks of acute and chronic graft-versus-host disease (GvHD) in about a few to 50 % [5] and may also suffer from late complications including solid tumours in about 10 % of cases [8]. These complications and persistent disease have a great impact on the quality of life of AA patients [10]. For many years recommendations had been to use HCT as a first line treatment in younger patients with severe disease (23) and IST for all other. Rarely treatment algorithms went beyond first line treatment recommendations.

In this single-center study, we analysed very long-term outcome of all patients with AA treated since 1973 by first-line HCT or IST, comparing not only survival, but also long-term response status and complications.

## Methods

### Study design and population

This retrospective cohort study included 302 patients diagnosed with AA who received HCT or IST as first-line therapy from 1973 to 2017 at the University Hospital Basel, Switzerland. Data included demographic information, pre-treatment blood values, date, type, source and protocols for therapy, response to therapy, status at last follow-up, causes of death and type of complications. Given the long observation period, many diagnostic methods had changed over time. Follow up was completed by 2019. Patients were followed at our center. Additional follow-up informations were obtained from referring centers.

### Diagnosis

Diagnosis and severity were defined by the Camitta Criteria [11]. For severe AA (SAA) the following criteria had to be fulfilled: bone marrow cellularity of < 30% and 2 of 3 of the following criteria: reticulocytes < 20 G/L (manual) or < 60 G/L (automated), absolute neutrophil count (ANC) < 0.5 G/L and platelets < 20 G/L. Patients with a neutrophil count < 0.2 G/L were classified as very severe AA (VSAA). Otherwise non severe AA (NSAA) was diagnosed [12] if there was no evidence for other disease causing marrow failure, hypocellular bone marrow was shown and cytopenias of at least two out of three peripheral blood cell lines below the normal values were documented: ANC < 1.2 G/L and ≥ 0.5 G/L, platelet count < 70 G/L, absolute reticulocyte count < 60 G/L, without fulfilling the criteria for SAA.

### Treatment

A total of 76 (25 %) patients received HCT as first line treatment, the first HCT was performed in 1973. Twenty nine patients (10%) received HCT as a second- or third- line treatment for relapse or non-response to immunosuppression. Treatment protocols and drug preparations changed over time; the majority of patients were conditioned with cyclophosphamide. After 1994 ATG (Thymoglobulin) was added. Fludarabine was given additionally since 2000 for older patients. Irradiation (total body, total lymphoid or thoraco-abdominal) was used in a minority of patients (n=4, 5.3%). GvHD prophylaxis consisted of methotrexate, combined with cyclosporine since 1979. Bone marrow was used as a primary source of stem cells (n=69, 90.8%), less frequently peripheral stem cells (n=5, 6.6%) or cord blood (n=2, 2.6 %). Mainly matched-related donor HCT was performed (n=62, 81.6%). Unrelated donor HCT was mostly second line treatment (n=24); 9 patients had unrelated donor HCT as part of first line treatment (7 of these had a congenital type of marrow failure). A total of 226 (75 %) patients received IST as first line therapy, of which the first course of ATG-based immunosuppression was given in 1973. Treatment protocols and drugs preparations changed over time: From 1976 to 1991 Lymphoser Berna (Swiss Serum and Vaccine Institute, Berne, Switzerland), from 1992 to 2006 Lymphoglobuline Merieux (Merieux, Lyon, France) and since 2007 ATGAM (Pfizer, New York, USA) was used as equine antithymocyte globulin. Besides, thirteen patients were treated with rabbit ATG (Thymoglobulin). From 1973 to 1981 thirty-two patients received haploidentical bone marrow infusion in addition to ATG, as previously described [13]. In none of these patients engraftment occurred and the hematopoetic reconstitution was autologous, thereby confirming that the therapy effect was due to IST alone [2]. These patients are counted among the IST group for the purpose of this analysis. Since 1981 methylprednisolone was given additionally to prevent serum sickness. Cyclosporine was added to the regimen in 1991 and was administered for at least one year. Norethandrolone, an androgen, was given until 1994. Few patients in the nineties were included in studies such as IL-3 or stem cell factor for relapsed disease, in the years 2002-2008 in the EBMT G-CSF trial [14] and since 2016 in the RACE trial (eltrombopag). Of the patients who received immunosuppression, 197 patients were never transplanted regardless of response. All patients received infectious disease prophylaxis and standard supportive care, including transfusions of red blood cells (since 1999 γ-irradiated (30 gy), leucocyte depleted) and platelet concentrates (since 2011 pathogen reduced (Intercept®)), as well as application of broad-spectrum intravenous antibiotics in case of fever and infection. They were either treated in laminar air flow rooms or in single rooms with reverse isolation [10].

### Response

Response was classified according to NIH criteria [15]. Complete response (CR) was defined by hemoglobin (Hb) levels > 100g/L, absolute neutrophile count (ANC) > 1.0 G/L, platelets > 100 G/L and no evidence of clonal evolution. Partial response (PR) was defined as transfusion independence, no longer meeting criteria for SAA and peripheral blood counts with Hb > 8 g/dl, ANC > 0.5 G/L and platelets > 20 G/L. Any patient not meeting any of the response criteria for PR or CR was classified as no response (NR).

### Relapse, Non-Engraftment and Graft failure

Relapse in the HCT group is defined as primary non-engraftment or late graft failure. In patients treated by IST relapse is defined as decreasing blood count counts requiring transfusions or administration of another course of IST respectively performing HCT in patients with a prior response (PR or CR) [14].

### Complications

Patients were evaluated regarding the following complications: clonal evolution to MDS/AML, secondary lymphoma and solid tumor, disease-related complications such as infections (bacterial, viral, fungal or protozoal), hemorrhage and thromboembolic events, therapy-related complications such as iron overload, cardiovascular events, endocrine dysfunctions, aseptic osteonecrosis and GvHD. Acute (aGvHD) and chronic GvHD (cGvHD) were diagnosed and graded according to published criteria [16, 17]. In case of hospital admission due to infection or hemorrhage, the event was classified as severe.

### Cause of death

Cause of death was subdivided in six categories: disease-related (infection or hemorrhage), treatment-related (GvHD, graft rejection or death during therapy time), clonal evolution (MDS or AML), secondary malignancy (solid tumor or lymphoma), other (no association to the disease) and unknown.

### Statistical analysis

The chi-square test was used for comparisons of categorial and the Mann-Whitney U-test for continuous variables among groups. OS was defined as time to death, event free survival (EFS) as time to death, time to relapse in responding patients or time to secondary clonal events. We defined current event free survival (cEFS) as the probability of being alive and in > partial remission even after several lines of treatment at the time of last treatment; e.g. a patient with IST, relapsing after response with HCT as second line treatment with long term survival with response, is considered a current event free survivor. Survival probabilities were estimated using the Kaplan-Meier estimator. The log-rank test was used to compare survival between patient groups. Cumulative incidence was used for relapse, secondary clonal complications and GvHD, with death of other causes as competing events, significance testing was by Fine and Gray. Data was analysed in the IBM SPSS Statistics 22 and STATA v15 for cumulative incidence.

## Results

### Baseline characteristics

Median follow up of surviving patients was 17 years (range: 1-41 years). Median age at diagnosis was 24 years (1-80 years), 18 years (1-66 years) in patients treated by HCT compared to 27 years (IQR 1-80 years) in the IST group. Sixty-nine patients (22.8%) were diagnosed with NSAA, 142 patients (47%) with SAA, 79 patients (26.2%) with VSAA and 9 (3%) with inherited bone marrow failure (of these 7 were transplanted upfront, in 2 diagnosis was made after failure of first line IST). Additional patient characteristics are shown in Table 1.

**Table 1:** Baseline characteristics

**Cases**	**All patients** **N=302**	**IST first-line** **N=226**	**Allo HCT first-line** **N=76**	**P-value**
**Age at diagnosis (years), median (IQR)**	24 (1-80)	27 (14-54)	18 (11-26)	< 0.001
**Sex (M/F) , n (%)**	161 (53) / 141 (47)	123 (54) / 103 (46)	37 (49) / 38 (51)	0.444
**Etiology of AA, n (%)**				0.014
**Idiopathic**	233 (77)	176 (78)	57 (75)	
**Congenital**	12 (4)	4 (1)	8 (11)	
**Toxic**	16 (5)	12 (5)	4 (5)	
**Drug-related**	19 (6)	16 (7)	3 (4)	
**Infectious**	22 (7)	18 (8)	4 (5)	
**Severity of AA, n (%)**				0.009
**VSAA**	79 (26)	60 (27)	19 (25)	
**SAA**	142 (47)	110 (49)	32 (42)	
**NSAA**	69 (23)	52 (23)	17 (22)	
**Inherited**	12 (4)	4 (2)	8 (11)	

### Therapy

First line treatment was IST in 226 patients (75%), HCT in 76 (25%) of patients. Many patients had second line and subsequent lines of treatment, e.g. IST was second line treatment in 70 (61%) and HCT in 18 (16%); some patients had HCT as third (n=14, 36%) or subsequent line of treatment. Overall, 103 of the 302 patients had a HCT during their treatment course. The third most common second or subsequent line of treatment was the use of eltrombopag (n=13; as part of second or third line treatment). Other treatment included growth factors (n=18), cyclosporine as single agent treatment (n=6) and eculizumab (n=2) to treat clinical paroxysmal nocturnal hemoglobinuria (PNH).

### Response

**Fig. 1:**
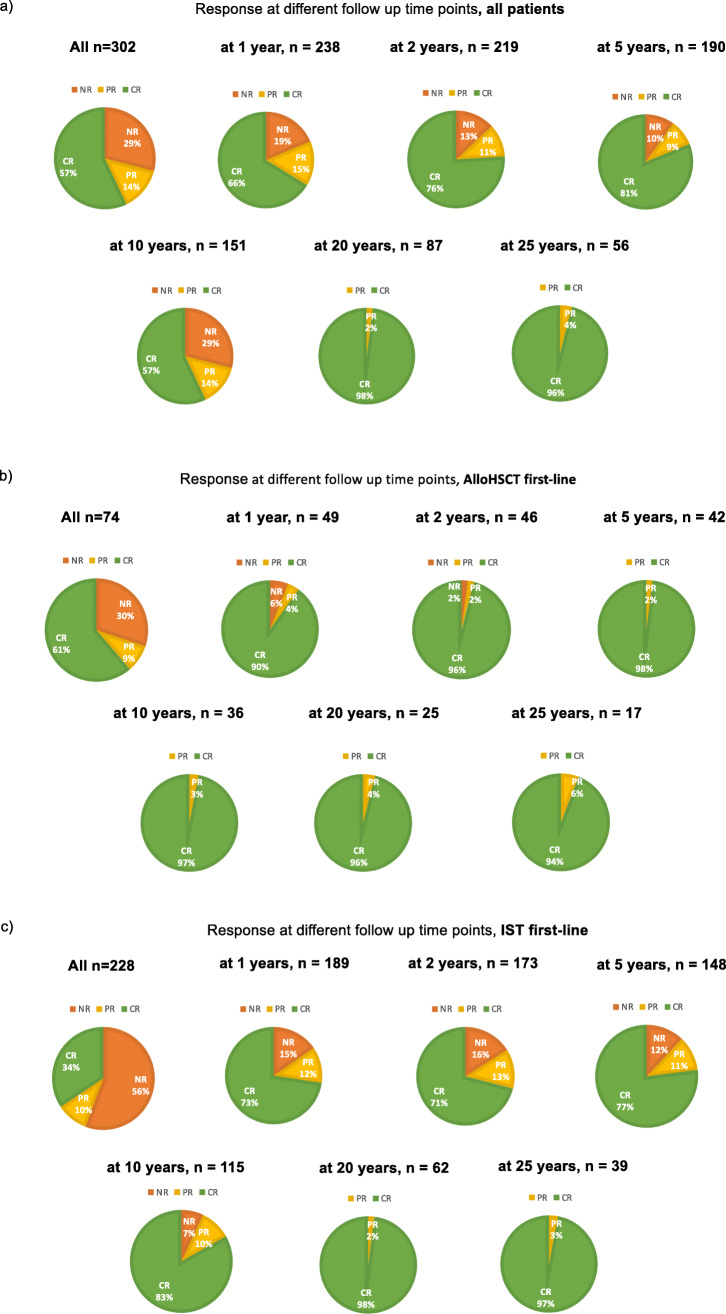
Pie graphs show percentage of response quality at different time points in follow up (a) in all patients b) in patients with first line HCT c) in patients with first line IST.

Figure 1 shows response to treatment over time defined as CR, PR and NR as described above. Pie graphs display the proportion of patients in the states of CR, PR and NR at 1 year up to over 25 years in surviving patients, separately for all patients and those with IST and HCT as a first line treatment. PR and NR occurred more frequently after IST as compared to HCT. With long term follow-up most surviving patients were in CR.

### Survival

**Fig. 2:**
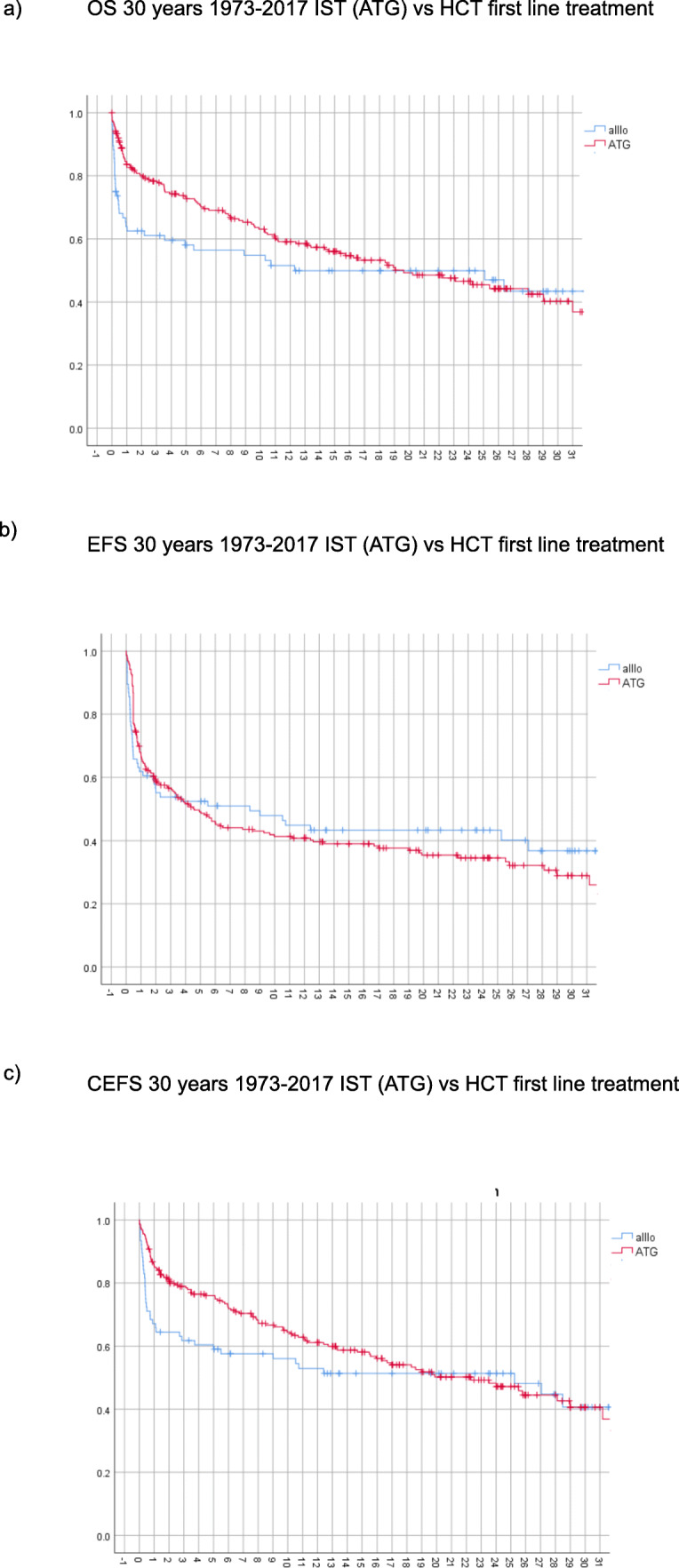
30 years survival (1973-2017) with respect to a) overall survival (OS) b) event free survival (EFS) c) current event free survival (CEFS)

Figure 2a outlines OS at 30 years separately for patients with IST and HCT as a first line treatment. EFS is shown in Figure 2b, whereas Figure 2c illustrates cEFS, i.e. the probability of being alive and in remission after several lines of treatment. CEFS is similar to OS indicating that only patients with functioning hematopoiesis remain alive long term. EFS curves show that only few events occur after 10 to 15 years. Table 2 lists OS, EFS, and cEFS at 30 years by first-line treatment, age, decade, and disease severity. There was a major improvement observed after 1980, whereas disease severity did not play an important role. The age distribution was atypical showing a higher mortality in children and adolescents due to higher proportions of children and adolescents being treated in the 1970s.

**Table 2:** Overall survival (OS), event free survival (EFS) and current event free survival (cEFS) at 30 years by age, decade, primary treatment and severity

	**OS**	**EFS**	**CEFS**
**Primary treatment**			
Allo HCT	43 (+ 14)%	37 (+ 13)%	41 (+ 15)%
IST	40 (+ 9)%	29 (+ 8)%	41 (+ 9)%
p-value	0.177	0.888	0.301
**Age**			
<18	34 (+ 14)%	32 (+ 10)%	32 (+ 15)%
18-40	51 (+ 13)%	35 (+ 11)%	49 (+ 13)%
40-60	32 (+ 21)% (at 25 years)	20 (+ 20)% (at 25 years)	33 (+ 24)% (at 25 years)
> 60	18 (+ 20)% (at 20 years)	30 (+ 18)% (at 15 years)	31 (+ 23)% (at 25 years)
p-value	0.049	0.597	0.096
**Decade**			
70s	22 (+ 12)%	18 (+ 12)%	22 (+ 12)%
80s	44 (+ 11)%	36 (+ 10)%	44 (+ 11)%
90s	51 (+ 18)% (at 20 years)	42 (+ 16)% (at 20 years)	27 (+ 39)%
00s	53 (+ 18)% (at 13 years)	27 (+ 16)% (at 13 years)	55 (+ 17)% (at 13 years)
10s	71 (+ 18)% (at 4 years)	50 (+ 18)% (at 5 years)	71 (+ 18)% (at 5 years)
p-value	0.003	0.084	0.021
**Severity**			
VSAA	15(+ 23)%	28 (+ 14)%	34 (+ 16)%
SAA	48 (+ 12)%	32 (+ 10)%	43 (+ 12)%
NSAA	41 (+ 14)%	34 (+ 14)%	21 (+ 31)%
p-value	0.073	0.623	0.190

Data presented as Kaplan Meier estimate and 95% confidence interval.

### Relapse and complications

#### Relapse

**Fig. 3:**
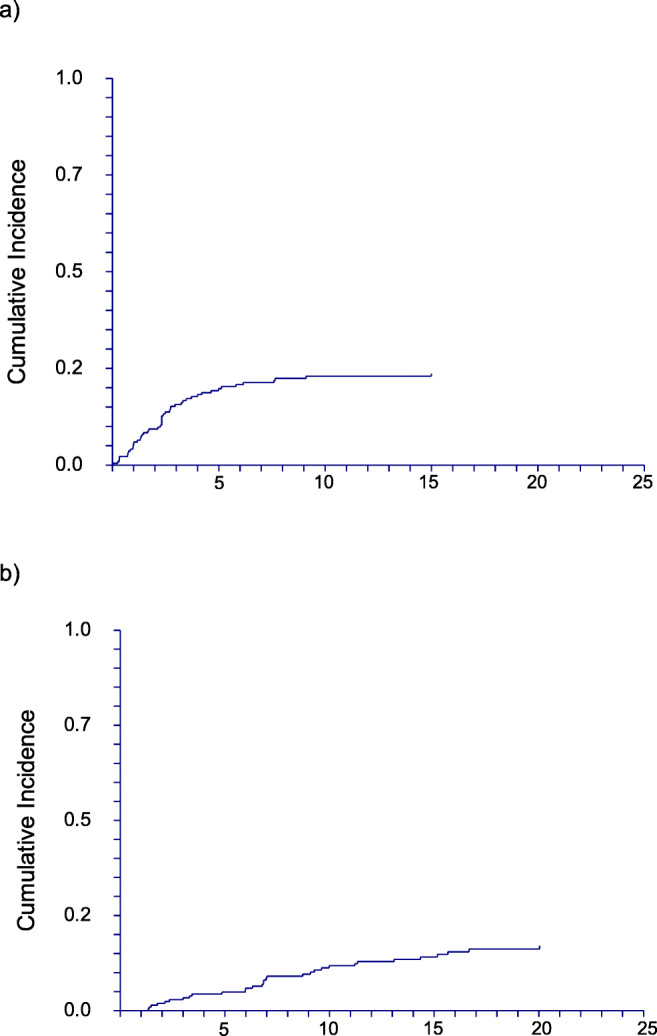
Cumulative incidence of a) relapse in IST patients b) MDS/AML occurrence

Figure 3a illustrates cumulative relapse incidence in patients with IST as a first line treatment with a PR or CR. Relapse incidence was 24 % (95% CI: 18-30) at 25 years after diagnosis, and of note, there were no relapse events 10 years after initial treatment. Among patients with HCT as a first or second-line treatment non-engraftment occurred in 11 patients and late graft failure in 4 patients. There was a tendency to fewer non-engraftment and graft failures in more recent decades, since 9 of 11 non-engraftment and 2 of 4 graft-failures occurred in 1970s and 1980s.

#### Clonal evolution

Figure 3b shows clonal evolution to secondary AML and MDS. The cumulative incidence was 13 % (95% CI: 10-18) at 25 years in all patients, 16 % (95% CI: 12-24) in patients with IST as a first line treatment; there seems to be no plateau, the last event occurring 20 years after treatment. In HCT patients, clonal evolution was rarely seen (1.3 %). Clonal evolution to MDS/AML was slightly higher in the years before 1990 but this difference did not reach statistical significance. Clinically overt PNH with florid intravascular hemolysis was diagnosed in 12 (4 %) cases during the course of disease.

#### Disease-related complications

Bleeding and infection were common (42.1% in the HCT and 56.2 % the IST group, respectively 89.5% and 90.7%, p=0.088 and 0.751), but its occurrence did not differ significantly between the two treatment groups (Table 3).

**Table 3:** Complications according to first- line therapy

	**HCT (76)**	**IST (226)**	**p-value**
**Hemorrhage**	32 (42.1)	127 (56.2)	0.088
**Severe hemorrhage**	18 (56.25)	63 (49.6)	0.751
**Infection**	68 (89.5)	205 (90.7)	0.343
**Severe infection**	40 (58.8)	105 (51.2)	0.203
**Thromboembolic event**	5 (6.6)	28 (12.4)	0.201
**Iron overload**	3 (3.9)	41 (18.1)	0.002
**Cardiovascular event**	1 (1.3)	24 (10.6)	0.011
**Endocrine dysfunction**	1 (1.3)	11 (4.9)	0.17
**Aseptic osteonecrosis**	2 (2.6)	6 (2.7)	0.991
**Acute GvHD Grade 2-4**	28 (36.8)		
**Resolved**	13 ()		
**Chronic GvHD**	25 (32.9)		
**Limited**	17 (68)		
**Extensive**	8 (32)		
**Resolved**	11 (44)		
**MDS/AML**	1 (1.3)	33 (14.6)	0.004
**Secondary malignancy**	8 (10.5)	17 (7.5)	0.411
**Solid tumor**	5	11	
**Lymphoma**	2	2	
**Other/unknown**	1	4	

#### Therapy-related complications

Iron overload was significantly more common in patients treated by IST (18.1 % versus 3.9 %, p=0.002) as well as cardiovascular events (10.6% vs. 1.3%, p=0.011). Acute GvHD Grade 2-4 occurred in 36.8% of the transplanted patients, of which 46.4% resolved over time. Fifty-five patients (72.4%) were at risk for cGvHD, of those 25 (32.9 %) developed cGvHD, of which 44% resolved.

#### Secondary malignancy

Secondary malignancy, mostly solid tumors but also a few cases with lymphoma, occurred in 10.5% of patients in the HCT group and in 7.5% of patients in the IST group (p=0.41).

### Cause of Death

During follow up 145 patients (48%) died (Figure 2). There were more early deaths in the HCT group and more late deaths in the ATG group. Most deaths in the HCT group occured within the first year after transplant, thereafter death rate remains more stable. Death was mainly therapy-related in the HCT group, whereas disease-related in the IST group (42.5% and 60%, p=0.026). In long-term survivors death was mainly due to malignancy (42.8%) in the HCT group, whereas in post-IST long-term surviving patients the majority of death were caused by typical chronic conditions in the elderly such as heart disease.

## Discussion

In this retrospective study we analysed long-term outcome of AA patients receiving first-line treatment with HCT or IST at our center since 1973. Considering the extensive study period over several decades and a median follow-up time of surviving patients of 17 years, we could gain insights on very long term survival of AA patients, therapy response and complications.

By this, we can report five major findings from our cohort: First, most deaths occured early and response obtained either through HCT or IST tended to be stable after five years, particularly in the second and third decade after treatment. Second, a considerable proportion of patients became long term responders and remained in remission long-term, with 96% of survivors at 25 years after first line treatment being in complete remission. HCT patients reached CR earlier whereas NR and PR was observed more often in IST patients, characterizing patients at risk for relapse. Since blood counts in IST treated patients more often do not return to normal and long-term cyclosporine maintenance therapy is required in a subgroup of patients [18], explained by the autoimmune character of the disease. Third, relapse most often occured within 5 years after IST, emphasizing that patients are most vulnerable during this period. Fourth, patients treated with IST are exposed to more complications, most importantly clonal evolution (MDS, AML), iron overload and cardiovascular events, whereas patients treated by HCT are almost exclusively affected by GvHD with, however a relatively low rate compared to historical data from HCT for malignant hematological diseases. Fifth, while death occurred early and was mainly therapy-related if HCT was first line treatment, patients treated by IST died more often later due to disease-related causes.

This study is unique in the very long term follow-up of AA patients treated since the 1970s. Although treatment strategies - particularly supportive care - has changed and outcome has considerable improved since the 1990s, this series can answer additional questions about long term prospects of these patients since the 1970s, given that a considerable propotion of patients is diagnosed at a young age. Over many years of follow-up the rate of severe late complications occuring in this population is overall moderate and focuses on very specific causes:

Clonal evolution to MDS/AML was seen more often in patients treated by IST than by HCT, with a rate (10 – 15 %) not higher than published previously [8, 9], but without a clear plateau during prolonged follow-up as shown in Figure 3b. Interestingly, we observed a trend to higher rates of leukemic transformation in the 1970s as compared to later periods. We assume that this trend is due to diagnostic uncertainty in this period, since the distinction of MDS and AA was less clear and possibly patients were diagnosed as AA who by todays standards (flow cytometry, cytogenetics, sequencing) would be considered hypoplastic MDS. Also the differentiation between acquired and inherited AA was less well established. We have complete data on secondary clonal evolution i.e. MDS, AML or hemolytic PNH. Unfortunately, we are lacking information on sequencing of myeloid neoplasia associated genes for most patients at long-term follow-ups. It would be highly interesting to test these patients during long-term follow up after IST in remission considering that this patient population with stressed hematopoiesis might show a different dynamic of clonal hematopoiesis and its potential associations (e.g. cardiovascular disease associated with specific driver mutations). We also do not present data on small, subclinical PNH clones as over the years diagnosis of PNH has evolved from HAM and Sucrose test to high sensitivity flow cytometric analysis.

Next to clonal evolution, IST as well as HCT treated patients showed an elevated risk for solid tumors [8, 9] emphasizing the importance of tumor surveillance not only in HCT but also IST treated patients. Tumor surveillance has been implemented in the screening recommendations for long-term survivors after HCT [19], but patients treated by IST are usually monitored less systematically and no consensus recommendations let alone guidelines exist on the topic of post-IST care and are therefore urgently needed.

Also the screening for iron overload and cardiovascular events in IST patients has not been addressed and compared with HCT patients so far. The increased rate of iron overload in our cohort could be explained by higher transfusion loads in IST patients, since response to therapy is often delayed and patients remain transfusion dependent for months after ATG. In addition, IST patients are at higher risk of relapse (11), leading to additional transfusions. At last, effective and tolerable iron chelation therapy had been introduced in recent decades [20] and may be limited by chronic renal failure, often occurring in patients with aplastic anemia treated by IST. It is of great importance to address this issue early since iron chelation therapy not only reduces iron overload but may also improve hematologic parameters in AA patients [21].

With respect to cardiovascular events, it is well-known that these occur at a higher rate in HCT patients than in the normal population [22]. However, data on cardiovascular outcome of IST treated AA patients is scarce. Our increased rate in comparison to HCT patients is in part explained by the older age of IST treated patients (median age of patients receivinig HCT as first line treatment: 18 (range 1-66) yrs versus 27 (1-80) yrs in patients with IST as first line treatment). Other reasons include long-term cyclosporine intake with the risk for dyslipidemia [23], less standardized follow-up care for cardiovascular disease in comparison to HCT patients, iron overload and remaining anemia.

Overall, the higher complication rate in IST patients (clonal evolution, secondary malignancy, iron overload, cardiovascular complications) emphasizes the need for outcome registries and consensus recommendations on the post-IST follow up care in AA patients and validate them thereafter.

Many studies have compared first line treatment with IST to HCT as an initial strategy [1, 2, 6, 24]. Our study additionally shows that patients may undergo several lines of treatment over the years and that a simple focus on first line treatment is not taking into account the longer term prospects of these patients. Patients may undergo up to 5 and 6 lines of treatment over time. Interestingly, looking at long-term survivors, a considerable proporption of patients stay in remission with CR after 25 years.

Several trials also have reported on the overall response to IST and HCT in AA [7, 25, 26]. In contrast, we focused on therapy response at last follow up, with the idea of describing long-term therapy outcome more realistically, and showing that the majority of patients – irrespective of treatment modality - reaches CR at last follow up. Conversely, responding patients become long-term survivors. These results may calm the endless discussion whether IST is inferior to HCT and only postpones the inevitable [5, 27].

Several limitations of the study merit consideration. The diagnosis criteria for AA have changed through the decades although the Camitta criteria had been defined already in the 1970 [11]. Nevertheless, distinction from hypoplastic MDS and from inherited bone marrow failures has evolved, and it cannot be excluded that misdiagnosis has confounded our results. We were also confronted by insufficient documentation or patients lost to follow-up due to this long observation period. Causes of death could not be determined in a considerable number of patients, in particular late deaths. Attribution of cause of death to either disease or treatment related becomes difficult as shown in patients such as UPN 2, who was treated in 1973 at the age of 44 years and died in 2018 in an old age home. Her blood counts had been normal in 2014 and had not tested thereafter.

In conclusion, the majority of AA survivors experience a good long-term survival and regain hematopoiesis irrespective of treatment modality, but patients treated with IST suffer from more complications and incomplete treatment response. To improve the outcome of IST patients consensus recommendations on post-IST follow-up care are urgently needed.

## Data Availability

The datasets generated during and/or analysed during the current study are available from the corresponding author on reasonable request.
